# Post-Delivery Milking Delay Influence on the Effect of Oral Supplementation with Bovine Colostrum as Measured with Intestinal Permeability Test

**DOI:** 10.3390/medicina56100495

**Published:** 2020-09-24

**Authors:** Maciej Hałasa, Dominika Maciejewska-Markiewicz, Magdalena Baśkiewicz-Hałasa, Krzysztof Safranow, Ewa Stachowska

**Affiliations:** 1 Department of Human Nutrition and Metabolomics, Pomeranian Medical University, 70-204 Szczecin, Poland; domi.maciejka@wp.pl (D.M.-M.); ewast@pum.edu.pl (E.S.); 2 Department of General Pathology, Pomeranian Medical University, 70-111 Szczecin, Poland; poziomka@pum.edu.pl; 3 Department of Biochemistry and Medical Chemistry, Pomeranian Medical University, 70-111 Szczecin, Poland; chrissaf@mp.pl

**Keywords:** bovine colostrum quality, biological activity, bioactive components, intestinal permeability, lactulose/mannitol absorption test

## Abstract

*Background and objective:* The health supplement bovine colostrum reportedly improves immunity and regulates intestinal homeostasis. Reliable assessment methods are needed to ensure the satisfactory biological activity of all marketed colostrum products. Of the well-established effects of colostrum use, the restoration of appropriate intestinal permeability assessed with the lactulose/mannitol (L/M) differential sugar absorption test upon supplementation with colostrum has been consistently observed. Milking time after delivery is one of the factors that influences the composition of bovine colostrum, which causes a rapid decrease in bioactive components. *Materials and methods:* We use the L/M test to evaluate the intestinal permeability reduction upon supplementation with colostrum (2 × 500 mg) harvested at various times after delivery (2, 24, and 72 h) or a placebo (whey). In our randomized, double-blind placebo-controlled (DBPC) trial, 31 healthy athletes were divided into four groups and assessed at baseline and after the intervention. *Results:* The trial revealed that only colostrum collected after 2 h and 24 h caused a significant reduction of intestinal permeability. The comparison of post-intervention vs. baseline Δ values produced statistically significant results for 2 h colostrum versus the placebo and 72 h colostrum groups. *Conclusions:* We conclude that the change of bovine colostrum composition over the first three days of lactation is accompanied by a decrease in its biological activity as measured with the L/M test. This test may offer a biological quality measure for colostrum.

## 1. Introduction

The increasing popularity of various plant or animal-derived substances used as natural food additives or health supplements is leading to the need for an evaluation system of these products [1]. This should be based on scientific medical methods that assess the actual value and safety of food supplements. Many kinds of these products have already been more or less thoroughly investigated, which has permitted the acceptance of some of them as safe and effective supplements. However, the standards that are required to be met by diverse suppliers, supposedly offering the same type of product often remain to be established [2]. The quality of food supplements made of natural products strongly depends on their source, the methods used to collect and handle them, and eventually the way they are preserved, stabilized, and packaged. An inappropriate approach to any of these elements may greatly deteriorate the expected biological activity of the final product. In effect, this may result in inconsistent evaluation feedback from the users, which may influence the perception of the entire group of products.

One of the most complex natural products that has repeatedly shown confirmed biological activity and beneficial influence on human health is bovine colostrum [3,4,5]. Since colostrum contains numerous relatively fragile bioactive components, including proteins and peptides, the risk that inadequate collection and handling procedures may negatively influence its quality is probably higher than with many other, less complex natural products [5,6]. Thus, it is very probable that some colostrum products entering the market may not be of the best quality. This is largely because clear and comprehensive quality standards for bovine colostrum products have not yet been established.

Several key points in the technological process determine the successful manufacturing of a clean, stable, and biologically effective market product based on bovine colostrum. Among them, three factors are of the most dramatic importance:

1. The hygiene standards of harvesting—the cleaner the collection, the fewer and the less aggressive manipulations are required to obtain a microbiologically clean final product;
2. The time of milking the colostrum—the sooner after delivery, the higher the content of biologically active products in colostrum;
3. The method used to obtain dehydrated product—the less aggressive thermic treatment used, the better the chance of preserving the bioactivity of the proteins and peptides in the product.

Establishing the quality of a biologically active product can be based on several approaches; one of these is the meticulous analysis of the content of the product in question molecule by molecule [7,8,9]. In the case of compound products, such as colostrum, this is very difficult to obtain. Moreover, even if it is successfully completed, the actual value of such a method can be questioned, as we know relatively little about the relationship between the precise composition of colostrum and its biological activity. Consequently, a much better way to establish basic standards regarding colostrum harvesting and handling in order to obtain a high-quality product seems to be the comparison of the biological activity of alternatively processed yields. To date, this has been attempted in vitro to test the proliferation-promoting activity of colostrum and milk at various stages of lactation on intestinal cell lines [10]. However, until recently, this approach was not possible in vivo in humans, as no methods have been available to assess the biological activity of bovine colostrum in simple, reproducible, and reliable tests.

According to traditional knowledge and literature, several potential biological effects of colostrum use that have been recognized in humans. Among them there are: various anti-infectious actions, including a direct antimicrobial effect and immune mechanism modulation; the reduction of inflammatory processes, including those responsible for the effector phase in immunopathology disorders likeautoimmunity or allergic diseases; and finally, the improvement of the healing processes [3,4,5]. Most of these beneficial results can potentially be directly linked to the gut’s homeostasis preservation, and more precisely, to the action of colostrum on the reduction of the permeability of the intestinal barrier. This effect of colostrum supplementation on the human intestinal barrier has been recently repeatedly confirmed [11,12,13]. Following the findings that colostrum use has a protective influence on increased permeability [14], we have also established that already increased permeability can be restored to normal values or at least markedly decreased upon supplementation with small doses of bovine colostrum [15].

The result of our previous experiment has proven that intestinal permeability testing based on differential sugar absorption can be regarded as a first widely available, reliable, and relatively simple indicator of the biological activity exerted by bovine colostrum in humans [15]. Consequently, in this trial, we used this tool to evaluate how the time of milking the colostrum may influence the extent of its bioactivity in humans, since we hypothesized that the longer the time from delivery, the lesser the expected biological activity of the product. This assumption was based on the well-established knowledge that the content of bioactive compounds in colostrum diminishes with time passing from delivery [4,16,17,18]. To increase the validity of our comparison, we have used colostrum from the same source, harvested and handled in precisely the same way; the only parameter that differed in the tested colostrum types was the milking time after delivery.

## 2. Materials and Methods

### 2.1. Recruitment

A group of 36 healthy volunteers—30 male and 6 female between 18 and 49 years of age (median = 35.8; mean = 34.5; standard deviation SD = 8.18)—was recruited from active athletes from various sport disciplines, including mixed martial arts (10), triathlon (11), cycling (9) and water polo (6). Their average BMI was 25.32 kg/m^2^ (mean = 25.27, SD = 3.31 kg/m^2^), and their mean weekly athletic training load was 407 min divided into 4.5 exercise units, which produced a mean metabolic equivalent of task (MET) equal to 13.3 MET-h/week.

Informed consent was obtained from each participant prior to enrolling in the study. All the participants claimed to undergo regular medical checkups and declared themselves to be fit for an intense athletic training regimen.

### 2.2. Study Design

#### 2.2.1. Allocation of Participants to Tested Groups and Initial Assessment: Day 0

**Figure 1:**
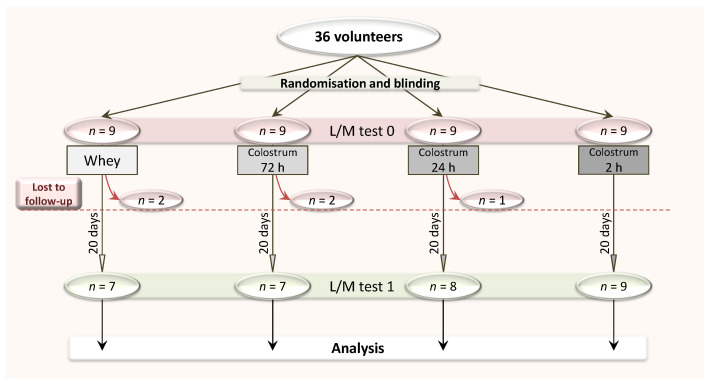
The outline of the study. L/M, lactulose/mannitol.

At the enrolment time (day 0), the 36 participants were randomly assigned (with the online tool https://www.randomizer.org/) to one of the four study groups receiving placebo (group 1) or colostrum milked at 72 h (group 2), 24 h (group 3) and 2 h (group 4) post-delivery. The study outline is presented in Figure 1. The administrator performed group allocation, but was not involved in laboratory procedures, while the investigators were blinded to group allocation. On day 0, the lactulose/mannitol (L/M) test (differential sugar absorption test) was performed for all participants to assess their initial intestinal permeability (details below).

#### 2.2.2. Supplementation: Days 1–20

Freeze-dried whole bovine colostrum obtained at 2 h, 24 h, and 72 h after calf delivery was kindly provided by Genactiv Sp. Z o.o., Poznań, Poland, packaged in unlabeled pouches (500 mg bovine colostrum of various milking times and 500 mg desiccated banana). While the 2 h colostrum was the regular stock Genactiv product, the company prepared the remaining two types (24 h and 72 h) for the purpose of the trial using an identical process of desiccation and packaging. A single dose of 500 mg of 2 h colostrum powder twice a day was shown to effectively restore appropriate intestinal permeability in our previous study [15]. Identical pouches used as placebo contained 500 mg of dehydrated whey and 500 mg of desiccated banana. This placebo, which was used in our previous study, was proven to have no statistically significant influence on intestinal permeability regulation. All colostrum used in the trial was desiccated in the freeze-drying process utilizing temperatures of no more than 40 °C, which allowed for the preservation of bioactive peptides and proteins. Conversely, the whey underwent a spray-drying process, where the air—reaching temperatures of 160 °C—may have potentially damaged the function of bioactive proteins.

The packages, including 40 pouches containing one of three types (2 h, 24 h or 72 h) of colostrum or placebo, were distributed to participants randomly assigned to groups by the trial administrator. The use of the provided test substances was scheduled to begin on day 1 of the trial in the twice-a-day mode—one pouch in the morning and one in the evening, with both doses taken 30 min before a meal—and lasting for 20 days. While using the tested substances, the participants were asked to take note of unusual or adverse reactions, including those from the digestive tract. If any occurred, to record them along with their general health status impressions in the original questionnaire provided for that purpose.

#### 2.2.3. Final Assessment: Day 21

After completing the supplementation period, we assayed the participants for their intestinal permeability on day 21 of the trial. This was performed again with the L/M test. Five of the originally included participants failed to attend the final assessment without stating a reason.

### 2.3. The Lactulose/Mannitol (L/M) Differential Sugar Absorption Test

Based on the difference in absorption pathways for lactulose and mannitol, the L/M test is the direct indicator of intestinal permeability. It measures lactulose absorption, which is possible only through the incompetent intestinal barrier. We performed the test according to the procedure described earlier [15]. Here, we provide the test principles in brief.

#### 2.3.1. Sugar Ingestion and Urine Collection

Participants were asked to perform the test in the morning following their regular exercise day. After an 8 h fast, each participant collected 100 mL of urine as a blank (negative control) sample and subsequently ingested 7.5 g lactulose and 2 g mannitol in 500 mL water. For the next 6 h, participants were asked to restrain from eating milk and dairy products, simple sugars, large doses of vitamin C and mannitol, and to collect all urine passed into one container.

#### 2.3.2. L/M Test Procedure

Aliquots of 400 μL of urine sampled from the collection container and from the blank sample were mixed with 40 μL of an internal standard (myo-inositol, 20 mg/mL) and lyophilized. Subsequently, we added 200 μL of anhydrous pyridine in hydroxylamine (25 mg/mL) to samples, mixed them, and heated them to 70 °C for 1 h; then, we centrifuged the mixture at 800× *g* for 5 min and collected 200 μL of supernatant. Then, we silylated the sugars with 100 µL of N-trimethylsilylimidazole for 30 min at 70 °C.

Finally, we assayed sugars by gas chromatography with an Agilent Technologies 7890A GC System and capillary column (15 m × 0.530 mm, 1.50 μm), (Supelco, Bellefonte, PA, USA). Chromatographic conditions were an initial temperature of 220 °C for 5 min, an increase at a rate of 10 °C/min for 2 min, an increase at a rate of 5 °C/min for 4 min and eventually an increase at a rate of 3.5 °C/min for 4 min to a final temperature of 274 °C, which was maintained for 7 min.

Hydrogen was the carrier gas. Lactulose, mannitol, and myo-inositol retention times in the samples were compared to those produced by commercially available standards.

### 2.4. Reference Limits

We established our own upper reference limit of the lactulose-to-mannitol ratio (L/M) of <0.035 based on literature [19]. We have used this limit in our previous published experiments [15,20].

### 2.5. Statistical Analysis

The statistical analysis was performed using Microsoft Excel 2016 (Microsoft, Redmond, WA, USA) and STATISTICA 13 (TIBCO Software Inc., Palo Alto, CA, USA). We verified the normality of the distributions of quantitative variables with the Shapiro–Wilk test, which showed that the distributions of L/M ratio values were significantly different from a normal distribution (*p* < 0.05) in 2 h colostrum and placebo groups. Therefore, we employed the nonparametric Wilcoxon signed-rank test to compare paired variables and the Kruskal–Wallis test followed by the Mann–Whitney U test to compare unpaired variables. Proportions were compared between groups using exact tests (Fisher’s exact test in the case of the 2 × 2 table). Differences with *p* < 0.05 were regarded as statistically significant, and these are indicated within the figures. We performed statistical power analysis using a normal distribution approximation, assuming the standard deviation of change between two subsequent L/M test results in the same patient was 0.015. The statistical power of our study with at least seven subjects in each group was sufficient to detect true differences in the change of intestinal permeability after supplementation between placebo and colostrum groups that were equal to 0.025 for the L/M ratio with 80% probability.

### 2.6. Bioethical Approval

We conducted the trial in accordance with the protocol conditions based on the KB-0012/127/18 approval, issued on 15 October 2018 by the Pomeranian Medical University Bioethics Committee.

## 3. Results

All participants except for one from the 2 h colostrum group reported full compliance with the supplementation schedule. A participant who failed to follow the administration schedule mistakenly received only one 500 mg dose of colostrum per day. His results were included in the study.

All of the volunteers participating in our trial were involved in intense athletic activities prior to and during the supplementation period, which was supposed to increase the intestinal permeability. Therefore, the results of their lactulose/mannitol differential sugar absorption tests were mostly either normal–high or above the reference range at the entry to the trial. The frequency of results that were over the reference limit at baseline ranged between 57% and 75% of participants across the trial groups; in the comparison of all four groups, no statistically significant difference was shown (Table 1).

### 3.1. Change of Intestinal Permeability upon Supplementation across the Groups

**Figure 2:**
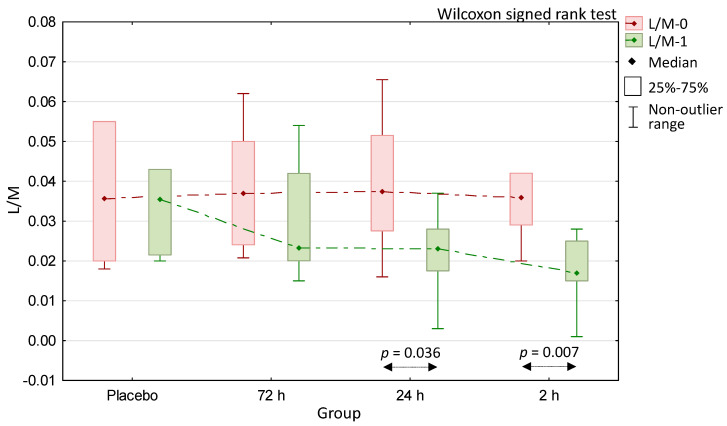
Comparison of lactulose/mannitol differential sugar absorption test results obtained before (L/M-0) and after (L/M-1) 20 days of supplementation with whey (placebo) and colostrum milked at 72 h, 24 h, and 2 h time points after delivery.

The comparison of the pre- and post-supplementation results within all of the trial groups revealed that neither in the placebo nor in the 72-h colostrum groups these differences reached statistically significant values (Figure 2). On the contrary, the two remaining colostrum-supplemented groups produced statistically significant results in this regard; these were for the 24-h colostrum group (*p* = 0.036) and for the 2 h colostrum group (*p* = 0.007). To assess the statistical significance value for these comparisons, we used the Wilkinson signed-rank test for paired samples.

### 3.2. Comparison of Intestinal Permeability Change (Δ) Due to Supplementation

**Figure 3:**
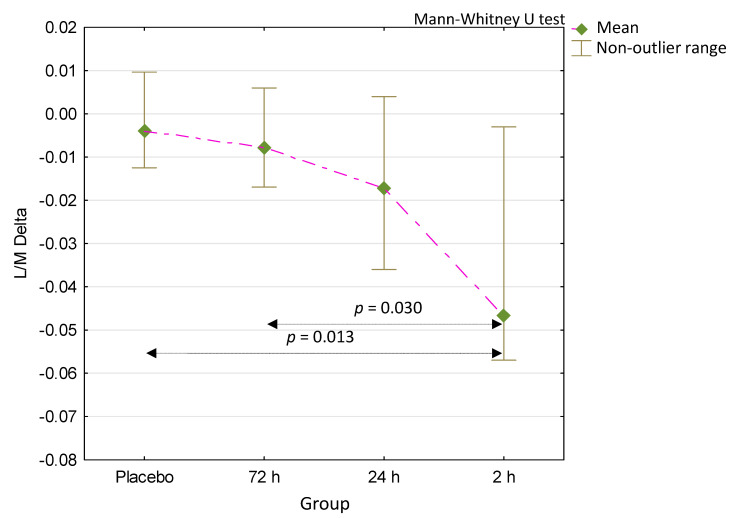
Comparison of the differences between the L/M-1 and L/M-0 test results (L/M Δ) reflecting the decrease in intestinal permeability after supplementation. *p* = 0.046 for comparison between four groups (Kruskal–Wallis test).

To compare the intestinal permeability-reducing effect of the supplementation administered in our trial, we calculated the post-intervention to the baseline L/M test results differences (L/M delta—Δ). There was a significant difference in L/M Δ values between the four groups (*p* = 0.046, Kruskal–Wallis test). Comparing the L/M Δ of all the colostrum-supplemented groups to the placebo group produced no statistically significant result except for the 2 h colostrum group (Figure 3). Comparing the L/M Δ from this group and the 72 h colostrum group also gave statistically significant results. The assessment of the comparison of L/M Δ values across all other groups produced no statistically significant effect. To test the statistical significance of the L/M Δ comparison results, we employed the Mann–Whitney U test for unpaired samples.

## 4. Discussion

Traditional knowledge, as well as modern scientific research methods, have helped to build a great body of evidence proving colostrum to be one of the most complete and rich natural sources of bioactive regulatory compounds [4,7,21,22,23]. However, despite its positive influences on both adolescent and adult human organisms, which have been demonstrated in numerous in vitro and in vivo studies, there are still users of colostrum who claim their experience with this supplement was not fully satisfactory. This could be due to the numerous products available on the market, some of which may not reach a sufficiently high-quality standard. Although these quality flaws may be of various origins, the milking time after the delivery of a calf seems to be of important influence on the final biological activity of colostrum-based products.

The analytical studies on the composition of colostrum and milk have shown that the concentration decrease of biologically active compounds found in the product of mammary glands is quite rapid and extensive [7,21]. The most dramatic drop in concentration can be observed for immunoglobulins and lactoferrin—two of the most abundant bioactive compounds of bovine colostrum [22]. The general protein content decrease ranged between 30% and 60% in colostrum milked 12 h from delivery, and between 35% and 70% when it was milked 24 h after delivery [16,24]. When specific bioactive compounds are analyzed, important reductions of immunoglobulins (IgG and IgA) and regulatory factors (TNF and IL-2) are observed already within the first 6–12 h [7].

These composition changes followed by a supposed biological activity decrease are the reason why traditional definitions of bovine colostrum formerly described it as a product of dairy cows obtained in the period between delivery and a 36 to 48 h post-calving [23]. Unfortunately, many makers of colostrum-based supplements do not clearly state the period within which they harvest their colostrum. Some brands of so-called colostrum supplements are made of 72 h and older collections—the time when most of the bioactive contents of colostrum are markedly decreased [4,14,22,24,25]. There are countries in which regulations prevent any harvesting, manufacturing, and marketing products containing colostrum obtained earlier than 120 h from delivery. In Japan, such milk that is taken five days or more after delivery is called “late colostrum” [26]. These regulations were intended to protect calves from being deprived of their natural supplementation, but the introduction of good cow breeding practices should prevent this [27]. Moreover, cows are mammals that are able to produce more colostrum than their calf may eat [28].

In our previous trials, we did not find any important relationships between the effect of colostrum supplementation and several anthropomorphic parameters, dietary habits, and pharmacological treatment [15]. In addition, we received no reports on side effects that could be attributed to colostrum supplementation in our trial participants, which was in accordance with literature reports [29]. Thus, we omitted the taking of a meticulous history and anthropomorphic measurement procedures from this trial. Moreover, this trial was aimed solely at finding the biological activity differences between various types of colostrum; the only information we collected from the participants was the potential complaints of any side effects, of which none were reported in this trial.

Our previous study proved the effectiveness of colostrum supplementation as an intestinal permeability regulator that is consistently able to restore the increased para-cellular transport in the gut mucosa to normal values [15]. In this study, we effectively used the supplementation dose of 2 × 500 mg, which is much lower than other authors have used [11,12,13,14]. Our dosage is compatible with the regimen proposed by several distributors in Europe, marketing colostrum as health-promoting supplement. Although athletes tend to use much higher doses of colostrum, our previous experiment has shown that this small dose can also be effectively used in physically active individuals [15]. The effectiveness of our small doses could be attributed to the fact that colostrum we used was prepared without treatment with high temperature (over 50 °C), which may be potentially damaging to some of the bioactive proteins, and thus, reduce part of the final product activity [30].

In or previous trials, we repeatedly evaluated the intestinal permeability with the lactulose/mannitol differential sugar absorption test, which is widely accepted as the most reliable intestinal permeability measurement technique [15,20,31,32]. Based on this, we designed this current experiment to use the L/M test for the assessment of the biological activity of various types of colostrum. This trial was also supposed to evaluate the L/M test as a useful tool for colostrum quality assessment. Since we expected the length of time between delivery and milking to strongly influence the variations in colostrum quality, we compared products identical in all regards except for this parameter in this trial.

We intended to perform our trial with individuals with relatively high para-cellular intestinal trans-epithelial transport to better observe the effect of the permeability reduction, due to colostrum supplementation. We expected that measuring this effect with the L/M test may be difficult when this kind of transport is very low at baseline. We also considered the possibility that colostrum most effectively downregulates the increased permeability. According to our expectations based on the literature and our previous reports [14,15,20,33], our participants—who were involved in intense athletic activity—had a baseline permeability that was at a relatively medium-high level, with the median being slightly above the reference limit level in all groups (Figure 2). The frequencies of results that were over the reference limit were also similar in all groups (Table 1).

A comparison of the baseline and post-intervention L/M test results has shown that the 2 h colostrum group produced a statistically significant decrease of intestinal permeability (Figure 2). When the delta (Δ) values resulting from the calculation of the difference between the post-intervention and baseline results were analyzed (Figure 3), the values produced by the 2 h colostrum group were of the largest magnitude (L/M Δ = 0.047). This group presented a statistically significant decrease of permeability as compared to the placebo (L/M Δ = 0.04) and the 72 h (L/M Δ = 0.08) colostrum groups (respectively, *p* = 0.015 and *p* = 0.030). Comparing the differences between all remaining groups showed no statistically significant difference. Furthermore, only in the 2 h colostrum group was any degree of permeability reduction observed in all participants, and this effect was the only such result that was significantly different from the placebo group (Table 1).

Despite producing a significant decrease of permeability between the baseline and post-intervention measurements (Figure 2), the 24 h colostrum has not proved to be statistically different from placebo or 72 h colostrum when L/M Δ results were compared (Figure 3). The 72 h colostrum group has brought the L/M Δ result of no significant difference from placebo (Figure 3), as well as it has shown no pre- to post-intervention difference of significant magnitude (Figure 2).

One of the limitations of our study was the lack of crossover protocol. The reason for this was the difficulty of introducing such a protocol in this multi-arm study, especially due to the relatively lengthy (at least two weeks-long) washout period required for studies with colostrum supplementation [11,12,14].

It is difficult to speculate which of the numerous bioactive components concentration decrease in colostrum is mostly responsible for its harvesting time-dependent drop of the biological activity observed in our trial. It is highly probable that the lessening of the excessive permeability results from several various colostrum-induced actions, including the reduction of zonulin release, anti-inflammatory effect, and improved healing [10,13,15,26]. It is difficult to predict if other important biological effects, such as an immunity boost, typically attributed to colostrum use actually deteriorate, due to delayed harvesting by the same degree as the permeability reduction effect. We can only speculate that this is highly probable, as at least some of the various biological effects of colostrum supplementation are supposedly caused by overlapping combinations of bioactive components [5,10,23]. There have also been links suggested between the ability to maintain intestinal homeostasis with its crucial component—intestinal permeability control—and the good immunity status of the entire organism [29,34,35,36].

This trial is the first experiment demonstrating how strongly the time of harvesting colostrum influences its biological activity. This should clear the way for the introduction of regulations obliging colostrum product manufacturers to state clearly the status of their product in this regard. Our findings may also help to improve the potential of assessing the quality of colostrum-based products, which currently is largely limited to compositional studies and purity tests, including microbiological safety checks [7,8,9,16,24].

Our experiment is also the first to show that assessing the quality of colostrum can be done through measuring the compound biological effect in humans. The L/M test utilized in our trial reflects the intestinal permeability status—one of the numerous biological traits influenced by supplementation with colostrum. However, it needs to be stressed that various health benefits resulting from supplementation with colostrum appear to be strongly interconnected with appropriate intestinal permeability [4,5,23,34,36,37,38,39]. Consequently, assessing the gut’s status in this regard provides us with information regarding broader implications than simply gastrointestinal tract homeostasis. The L/M test also seems to be a superior indicator than measuring cytokines and other regulatory molecule concentrations or changes in the proportional composition of leucocyte populations in the blood. Such methods were employed in the past, but the limited reproducibility of these attempts did not permit their introduction as acceptable indicators of the biological activity of colostrum. The advantage of the L/M test for that purpose is not limited to the good reproducibility of its results; it also comes from the fact that the change in the permeability measurement is the result of a complex series of biological events and not simply the change of a single parameter, such as the regulatory molecule concentration [15,20,37,40,41].

Additional studies will be required to optimize the timing of the collection of colostrum. Additionally, further thorough studies are required to establish the relationship between the composition of colostrum at various post-partum time stages and its biological activity. Other than the time of collection, parameters that possibly influence the quality of colostrum products should also be considered in these studies. One of these attempts, presenting not only very detailed compositional studies, but also relating them to a rat-based bioactivity assay, has been presented most recently by Playford et al. [42]. The conclusions from this work generally stand in accordance with our results.

## 5. Conclusions

We can conclude that when colostrum types differing from each other exclusively by milking time are compared, the best of them (in terms of inducing a reduction of intestinal permeability) is the colostrum collected within 2 h from delivery. In addition, the results show that the measured biological activity of colostrum milked at 72 h after delivery appears to be very little different from that caused by a placebo. The practical application of these trial results may help to establish a novel approach to the quality appraisal of colostrum-based products by assessing one important aspect of their biological activity with the L/M test.

## Figures and Tables

**Table 1:** The number and proportion of participants whose test results prior to (L/M-0) and after (L/M-1) the supplementation were equal to or above our own reference limit of L/M ≤ 0.035. The number and proportion of participants whose intestinal permeability decreased after supplementation (L/M Δ = L/M-1 − L/M-0 < 0) is also presented. The *p*-value for differences in the proportion of results above the reference limit between four groups was calculated with the exact test.

	L/M-0 Equal or Above Reference Limit	L/M-1 Equal or Above Reference Limit	L/M Δ <0
Placebo	4	4	3
*n* = 7	57%	57%	43%
Colostrum 72 h	5	3	4
*n* = 7	71%	43%	57%
Colostrum 24 h	6	1	6
*n* = 8	75%	13%	75%
Colostrum 2 h	6	1	9
*n* = 9	67%	11%	100% *
*p*-value (exact test for 4 × 2 table)	0.95	0.15	0.049

* *p* = 0.019 vs placebo group (Fisher’s exact test).
